# Application of Reverse Micelle Sol–Gel Synthesis for Bulk Doping and Heteroatoms Surface Enrichment in Mo-Doped TiO_2_ Nanoparticles

**DOI:** 10.3390/ma12060937

**Published:** 2019-03-21

**Authors:** Roberto Nasi, Serena Esposito, Francesca S. Freyria, Marco Armandi, Tanveer A. Gadhi, Simelys Hernandez, Paola Rivolo, Nicoletta Ditaranto, Barbara Bonelli

**Affiliations:** 1 Department of Applied Science and Technology, Corso Duca degli Abruzzi, 24 I-10129 Torino, Italy; roberto.nasi@polito.it (R.N.); serena_esposito@polito.it (S.E.); francesca.freyria@polito.it (F.S.F.); marco.armandi@polito.it (M.A.); simelys.hernandez@polito.it (S.H.); paola.rivolo@polito.it (P.R.); 2 U.S. Pakistan Center for Advanced Studies in Water (USPCAS-W), Mehran University of Engineering and Technology, Jamshoro 76062, Pakistan; tanveer.polito@gmail.com; 3 Center for Sustainable Future Technologies—CSFT@POLITO, Istituto Italiano di Tecnologia, Via Livorno 60, 10144 Torino, Italy; 4 Department of Chemistry, Università degli Studi di Bari “Aldo Moro”, via Orabona 4, 70125 Bari, Italy; nicoletta.ditaranto@uniba.it; 5 INSTM Unit of Torino-Politecnico, Politecnico di Torino, Corso Duca degli Abruzzi, 24 I-10129 Torino, Italy

**Keywords:** TiO_2_ nanoparticles, Mo-doping, band-gap

## Abstract

TiO_2_ nanoparticles containing 0.0, 1.0, 5.0, and 10.0 wt.% Mo were prepared by a reverse micelle template assisted sol–gel method allowing the dispersion of Mo atoms in the TiO_2_ matrix. Their textural and surface properties were characterized by means of X-ray powder diffraction, micro-Raman spectroscopy, N_2_ adsorption/desorption isotherms at −196 °C, energy dispersive X-ray analysis coupled to field emission scanning electron microscopy, X-ray photoelectron spectroscopy, diffuse reflectance UV–Vis spectroscopy, and ζ-potential measurement. The photocatalytic degradation of Rhodamine B (under visible light and low irradiance) in water was used as a test reaction as well. The ensemble of the obtained experimental results was analyzed in order to discover the actual state of Mo in the final materials, showing the occurrence of both bulk doping and Mo surface species, with progressive segregation of MoO_x_ species occurring only at a higher Mo content.

## 1. Introduction

Titanium dioxide (TiO_2_), in virtue of its chemical stability and low toxicity, is currently used in a wide range of applications, from dye-sensitized solar cells, to sensor devices and paints [1,2,3]; most of all, TiO_2_ is one of the most investigated photocatalysts (along with ZnO), as it is able to decompose several organic/inorganic pollutants in both liquid and gas phases [4,5,6].

TiO_2_ may occur as anatase, rutile, or brookite phases; amorphous TiO_2_ may be obtained, as well, and by properly tailoring the synthesis conditions, pure or mixed phases are obtained. While in bulk TiO_2_, rutile is the stable phase above 600 °C, synthesis methods starting from solutions containing a Ti precursor generally lead to the formation of anatase. When TiO_2_ nanoparticles (NPs) are obtained, polymorphs that are usually metastable in the bulk may be stabilized instead. Among TiO_2_ polymorphs, anatase has the lowest surface energy, and NPs of 15–30 nm dimension are easily obtained [7,8,9], for instance, from alkoxide precursors. However, if a strong acid is added, brookite NPs are more likely to form [10,11].

As far as photocatalytic applications are concerned, anatase has a band-gap (E_g_) of 3.2 eV, whereas that of rutile is 3.0 eV and that of brookite is in the range between 3.1 and 3.4 eV [12,13,14]: consequently, TiO_2_ is mainly active under UV light. Moreover, electron-hole pairs are characterized by a relatively fast recombination rate (from pico- to micro-seconds) [15] that decreases its photocatalytic efficiency. In addition, the TiO_2_ polar surface has a poor adsorption ability towards non-polar organic pollutants, further limiting its efficiency towards the removal of organic pollutants [16].

With the aim of improving TiO_2_ photocatalytic activity, doping with heteroatoms, including transition metals (Cr, Co, Fe, Ni, Mn, V, Cu, Ni, and Zn) and non-metals (vide infra), has been subject to many studies, since it should allow narrowing the band gap and improving its solar light absorption [17,18,19,20]. Concerning doping with transition metals, the absorption edge shifts towards longer wavelengths due to charge-transfer transitions between *d* electrons of the transition metals and the CB (conduction band) or the VB (valence band). Rare earths have good electron trapping properties, resulting in a large absorption edge shift towards longer wavelengths, whereas defects chemistry can also play an important role in the reactions kinetics and charge recombination.

Non-metals (C, B, I, F, S, and N) doping at the O sites of TiO_2_ NP_S_ have also been largely exploited: N appears to be one of the most efficient (and investigated) dopants, the enhancement of charge separation being ascribed to the formation of paramagnetic species [O–Ti^4+^–N^2−^–Ti^4+^–V_O_], where V_O_ is an oxygen vacancy. With non-metals doping, impurity states are located near the VB edge and their role as recombination centers might be minimized [17,21].

Other studies show, instead, that if TiO_2_ is modified by adding noble metals (such as Ag, Pt, Pd, Rh and Au), electron-hole recombination is hindered by the resulting Schottky barrier at the metal–TiO_2_ interface, where the former acts as a storing-transporting mediator of photogenerated electrons from the surface of TiO_2_ to an acceptor (in the reaction medium). Consequently, the photocatalytic activity increases as the charge carrier recombination rate decreases [22,23,24,25,26].

Concerning transition metals, Mo doping is very promising since it introduces an empty donor level below the CB (n-type doping), which only slightly perturbs the band [27], so avoiding strongly localized *d* states that can reduce the mobility of charge carriers [28].

High surface area NPs, with inter-and/or intra-particle porosity, can be obtained by using either hard or soft templates. Concerning soft-templates, mesoporous TiO_2_ particles are obtained by using either a triblock copolymer (e.g., Pluronic P123) or an ionic surfactant (e.g., cetyltrimethylammonium bromide), whereas when di-block copolymers are used (e.g., Brij-n), porosity forms among NPs [29].

Out of the different syntheses reported in the literature, the reverse micelles method proposed by Chandra et al. [30] allows obtaining high surface area Mo-doped TiO_2_, SiO_2_ and ZrO_2_ NPs where, simultaneously, Mo is fairly dispersed. With such a method, the self-assembly of surfactant molecules in the organic phase produces a “nanoreactor” where reactions in water phase take place; the size of the so-obtained reverse micelles, tunable through the polar head group and the alkyl tail length and structure, allows controlling both size and shape of the NPs [31]. The reverse micelle core provides a suitable environment for the controlled nucleation and growth of TiO_2_ NPs, simultaneously affording a good dispersion of MoO_x_ species.

Here, we report the synthesis and the physico-chemical properties of a set of pure and Mo-doped TiO_2_ NPs, obtained by properly modifying the method reported by Chandra et al. and adapting it to TiO_2_ [30]. Here, different Mo contents were studied and also a different surfactant was employed, namely polyoxyethylene (20) oleyl ether, characterized by the presence of a double bond and a different molar mass, as both properties can affect the size of the reverse micelles and, consequently, that of the obtained NPs.

The samples properties were characterized by means of a set of techniques (X-ray powder diffraction, micro-Raman spectroscopy, N_2_ adsorption/desorption isotherms at −196 °C, energy dispersive X-ray analysis coupled to field emission scanning electron microscopy, X-ray photoelectron spectroscopy, diffuse reflectance UV–Vis spectroscopy, and ζ-potential measurement.), and the photocatalytic degradation of the model dye Rhodamine B under visible light was used as a test reaction to gain insights into the state of Mo in the materials.

## 2. Materials and Methods

All reagents were from Sigma-Aldrich (Milan, Italy), if not otherwise specified.

Pure and Mo-doped TiO_2_ NPs were prepared by sol–gel reverse micelle microemulsion method [14], where polyoxyethylene (20) oleyl ether (Brij O20) was the surfactant, cyclohexane the oil phase, titanium(IV) butoxide 97% (Ti-(BuO)_4_) and ammonium heptamolybdate tetrahydrate ((NH₄)₆Mo₇O₂₄*4H₂O, purum p.a. ≥ 99.9%) the Ti and Mo precursors, respectively.

Proper precursor amounts were used in order to obtain nominal contents of 0.0, 1.0, 5.0 and 10.0 wt.% Mo/(Mo+TiO_2_). A typical synthesis involves the following steps: the surfactant is dispersed in cyclohexane by stirring at 50 °C, while the salt precursor is dissolved in MilliQ water at the same temperature. Afterwards, the salt solution is added to the oil/surfactant mixture and stirred for 45 min, with formation of a water in oil (w/o) microemulsion of surfactant nanoreactors. Ti-(BuO)_4_ is then added dropwise to the emulsion. The mixture is stirred for 2 h at the constant temperature of 50 °C, and finally the emulsion is broken by addition of 2-propanol, followed by sonication. The solid phase is collected by centrifugation and dried at 100 °C for 24 h, followed by calcination in air at 500 °C for 2 h with a temperature ramp of 2.5 °C/min to remove the surfactant.

X-Ray Diffraction (XRD) patterns were obtained by means of X’Pert Phillips diffractometer operating (Phillips-PANalytical, Almelo, The Netherlands) at 40 kV and 40 mA equipped with Cu Kα radiation (step scan = 0.02 2θ, time per step = 2 s).

Raman spectra were acquired on a Renishaw InVia Reflex micro-Raman spectrometer (Renishaw plc, Wotton-under-Edge, UK) equipped with a cooled Charge-Coupled Device (CCD) camera. The Raman source was a diode laser (λ_ex_ = 514.5 nm), and the inspection occurred over pelletized samples to ensure a “flat” surface, through a microscope objective (100X), in backscattering light collection. The following conditions were employed to collect each spectrum: 0.5 mW laser power, 10 s of exposure time and 1 accumulations.

N_2_ adsorption/desorption isotherms at −196 °C were obtained on a Micromeritics ASAP 2020Plus instrument (Micromeritics, Norcross, GA, USA) on powder samples previously outgassed at 120 °C for 1 h (temperature ramp = 5 °C/min), followed by an isothermal step of 2 h at 150 °C. The sample’s specific surface area (SSA) was calculated according to the Brunauer–Emmett–Teller (BET) method; pore total volume was measured at p/p^0^ = 0.99; pore size distribution was determined by applying the BJH (Brunauer–Joiner–Hallenda) method to the isotherm desorption branch.

Field Emission Scanning Electron Microscopy (FESEM) micrographs were taken on a ZEISS Supra 40 FESEM instrument (Carl-Zeiss AG, Oberkochen, Germany) equipped with an Energy Dispersive X-ray (EDX) probe used to determine the actual Mo/Ti atomic ratio, to be compared to nominal value, by a raster scan of ~0.05 mm^2^ of sample surface. For both FESEM and EDX analysis, a small amount of powder was pressed on the sample holder (a carbon conductive adhesive tape) without any conductive coating. FESEM micrographs were then analyzed by using the ImageJ software (version 1.50i), by arbitrarily selecting three parallel lines on the micrographs and by measuring particle edge-to-edge dimension, so obtaining the average particle size listed in Table 1.

X-ray Photoelectron Spectroscopy (XPS) analyses were performed with a Versa Probe II Scanning XPS Microprobe spectrometer (Physical Electronics GmbH, Ismaning, Germany). The measurements were done with a monochromatised Al Kα source (x-ray spot 100 μm), at a power of 24.8 W. Wide scans spectra were acquired in Fixed Analyzer Transmission (FAT) mode with a pass energy of 117.40. An electron gun was used for charge compensation (1.0V 20.0 μA). Data processing was performed by using the MultiPak software v. 9.8.0.19.

Diffuse reflectance (DR) UV-Visible (UV-Vis) spectra of the powders were recorded in the 200–800 nm range by using a UV–Vis Varian Cary 5000 spectrophotometer (Varian Instruments, Mulgrave, Australia) equipped with an integration sphere. The DR spectra is reported as Kubelka–Munk function (Equation (1)) where *R* is the absolute reflectance of the layer, *s* the scattering coefficient, and *k* the molar absorption coefficient under the assumption of “infinite” thick layer.

(1) $$F\left(R\right)=\;\frac{1-R^{2}}{2R}\;=\;\frac{k}{s}$$

ζ-potential was measured on a Zetasizer Nano ZSP apparatus (Malvern Instruments Ltd., Malvern, Worcestershire, UK) on suspensions of 5 mg sample in 10 mL distilled water. Before the measurement, the suspension was sonicated for 5 min and pH was adjusted by using either HCl or NH_4_OH. The corresponding ζ-potential was obtained from the electrophoretic mobility, according to the Smoluchowski’s approximation.

To evaluate the photocatalytic response of the samples, Rhodamine B (RhB) was used as a model dye pollutant, the details of the photocatalytic evaluation and reactor being reported elsewhere [33]. In brief, a 9-Watt white fluorescent lamp (with maximum 3.00% UV portion) with very low irradiance (33 W/m^2^, to minimize dye sensitization effect) was used as visible light source (400–700 nm) to irradiate the dye solution containing 40 mg of synthesized sample and 50 mL of RhB dye at a concentration of 5 ppm.

## 3. Results and Discussion

### Physico-Chemical Characterization of the Prepared Samples

The sol–gel chemistry of metal alkoxides is to some extent ruled by the rapid hydrolysis of the alkoxy groups, which explains why careful handling and storage of such reagents are required [34,35]. The vigorous reaction of Ti alkoxides with water leads to (undesired) formation of Ti oxo/hydroxo precipitates: the reverse micelles method is useful to control the rate of hydrolysis and condensation by the frequency of intermicellar exchanges and by the water content in the micelle core, in addition to the structure-directing role of the surfactant. The surfactant indeed provides a cage-like environment that limits the nucleation, growth, and agglomeration of the particles, which dimensions depend on the water to surfactant molar ratio and surfactant composition and morphology.

As reported by Wang et al. [36] and Chandra et al. [30] the size of reverse-micelle core depends on the synthesis parameters, predominantly the water to surfactant ratio and the nature of the solvent medium. Nevertheless, both size and shape of the NPs can be tuned by changing the polarity of hydrocarbon chain and the number polyoxyethylene groups. For instance, the non-ionic surfactant Brij-58 (linear formula C_16_H_33_(CH_2_CH_2_O)_20_OH; molar mass ~1124 g/mol) allows obtaining TiO_2_ NPs with an average size of about 23 nm [36]. In this study, instead, another surfactant was used, namely the Brij-98/O20 (linear formula C_18_H_35_(OCH_2_CH_2_)_20_OH; molar mass ~1150 g/mol): our purpose was to investigate the effect of another moiety on the size of the final NPs and on the overall Mo dispersion.

**Figure 1:**
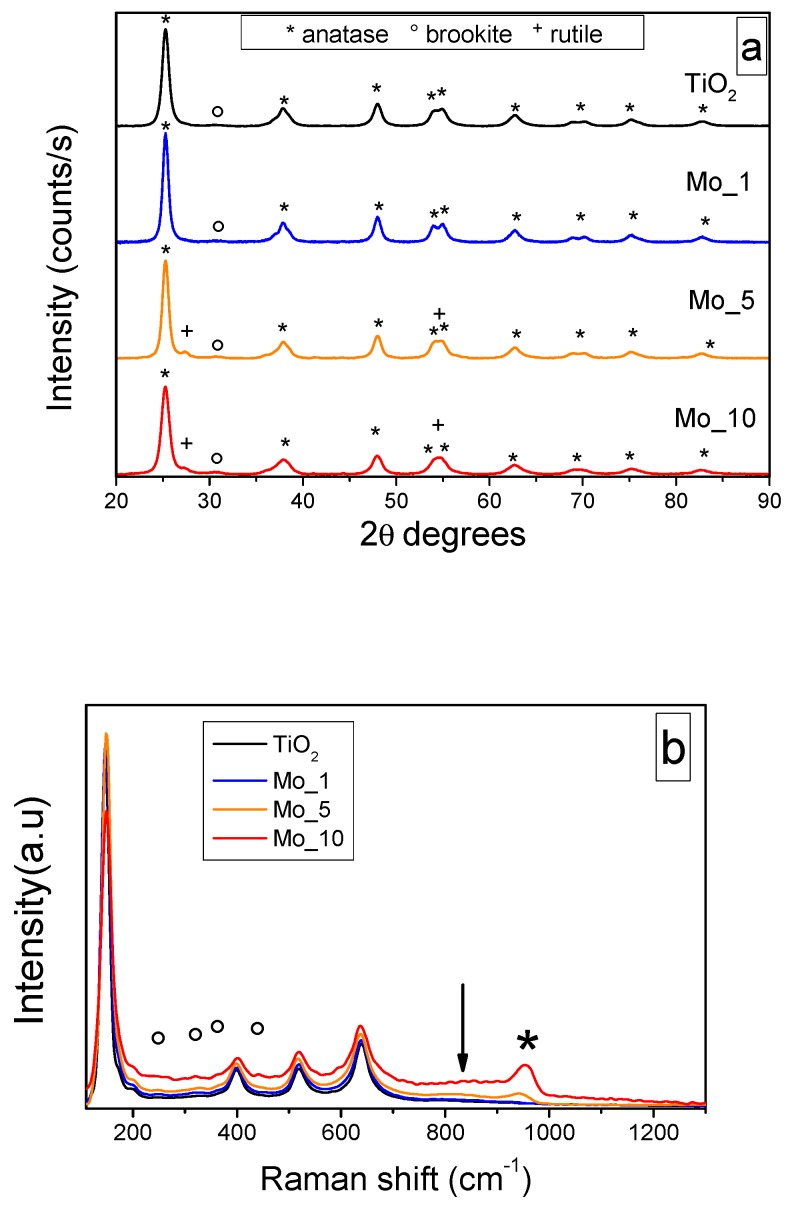
XRD patterns (**a**) and Raman spectra (**b**) of the studied powder samples (Phase symbols: * anatase; ^o^ brookite; ^+^ rutile).

Figure 1a reports the powder XRD patterns of the synthesized samples in the 20–90 2θ range: in agreement with the calcination temperature, the TiO_2_ sample showed the main peaks of anatase (labelled by asterisks in the Figure, at the 2θ values of 25.2 (011), 37.8 (004), 47.9 (020), 53.8 (015), 54.9 (121), 62.6 (024), 68.7 (116), 70.1 (220), 74.9 (125), and 82.5 (224)), along with an additional broad and weak peak centered at ca. 30.7 2θ (circle) that can be ascribed to the (121) diffraction of brookite. The formation of such a phase, likely occurring in small amounts, can be assigned to the adopted synthesis. The XRD patterns of the Mo_1 sample did not differ much from those of TiO_2_, whereas with both Mo_5 and Mo_10 samples, two additional peaks (cross) at 27.2 and 54.4 2θ values are respectively assigned to the (110) and (211) diffraction peaks of rutile. Since the ionic radius of Mo^6+^ ion (0.059 nm) is very close to that of Ti^4+^ ion (0.0605 nm), Mo doping in TiO_2_ mostly occurs by substitution, with formation of impurities/defects. It is generally acknowledged that the structure of rutile is more tolerant to defects than that of anatase and so, based on the current XRD results, it can be inferred that the formation of rutile was favored at higher Mo content [37,38]. The Williamson–Hall method was used to calculate the crystallite sizes reported in Table 1: showing a slight increase with Mo_1 and Mo_5 samples. No signals ascribable to MoO_x_ phases were detected, even at the highest Mo content: such a result indicates that Mo-containing phases, if present, are likely very well dispersed and cannot be detected by XRD.

The Raman spectra in Figure 1b basically confirm the XRD results: with all the samples, the Raman modes of anatase are observed at 147 (E_g_), 199 (E_g_), 399 (B_1g_), 519 (B_1g_) and 639 (E_g_) cm^−1^. With the Mo_5 and Mo_10 samples, the band at 639 cm^−1^ is slightly red-shifted (637 cm^−1^) with respect to that of bare TiO_2_: the most intense mode of rutile (A_1g_) occurs indeed at 636 cm^−1^ and so the observed shift could be ascribed to the simultaneous presence of both rutile and anatase, in agreement with the corresponding XRD patterns. Additional Raman signals (circles in Figure 1b) were observed at 246, 327, 362, and 448 cm^−1^, their intensities increasing with the Mo content: they are assigned to the A_1g_ (246 cm^−1^), B_1g_ (327 and 448 cm^−1^), and B_2g_ (362 cm^−1^) modes of brookite. The most intense band of brookite (B_1g_ mode) was usually found at 152 cm^−1^ and, here, is likely superposed to that of anatase. Indeed, the maximum of the main peak was blue-shifted with the Mo_10 sample, indicating a strong interaction of the heteroatoms with the TiO_2_ matrix.

With the Mo_5 and Mo_10 samples, two additional Raman signals were observed: a band occurring at 944 cm^−1^ with the Mo_5 sample and at 956 cm^−1^ with the Mo_10 sample (asterisk), and a broad signal occurring in the 760–880 cm^−1^ range of the Mo_10 spectrum (arrow). The former signal was assigned to Mo=O groups stretching [39,40]: with systems where a comparable amount of MoO_3_ was supported on TiO_2_ by incipient wetness impregnation, a similar band was observed in the 934–954 cm^−1^ range, shifting to higher wavenumbers with the Mo content, and having a broad and asymmetric shape, as here. The band position suggested the presence of Mo_7_O_24_^6−^ or Mo_8_O_26_^4−^ species, where Mo is octahedrally coordinated. Tetrahedral hydrated MoO_4_^2−^ species (that should give a Raman band at 934 cm^−1^) were not observed here, even at the lowest Mo content, indicating that Mo- doping is mainly related to the TiO_2_ bulk, whereas at higher Mo contents, formation of polymolibdate species takes place at the surface of the NPs. The broad Raman signal in the 760–880 cm^−1^ range was ascribed to the Mo–O–Mo bond of the same Mo_7_O_24_^6−^ or Mo_8_O_26_^4−^ species, in agreement with the literature [41].

**Figure 2:**
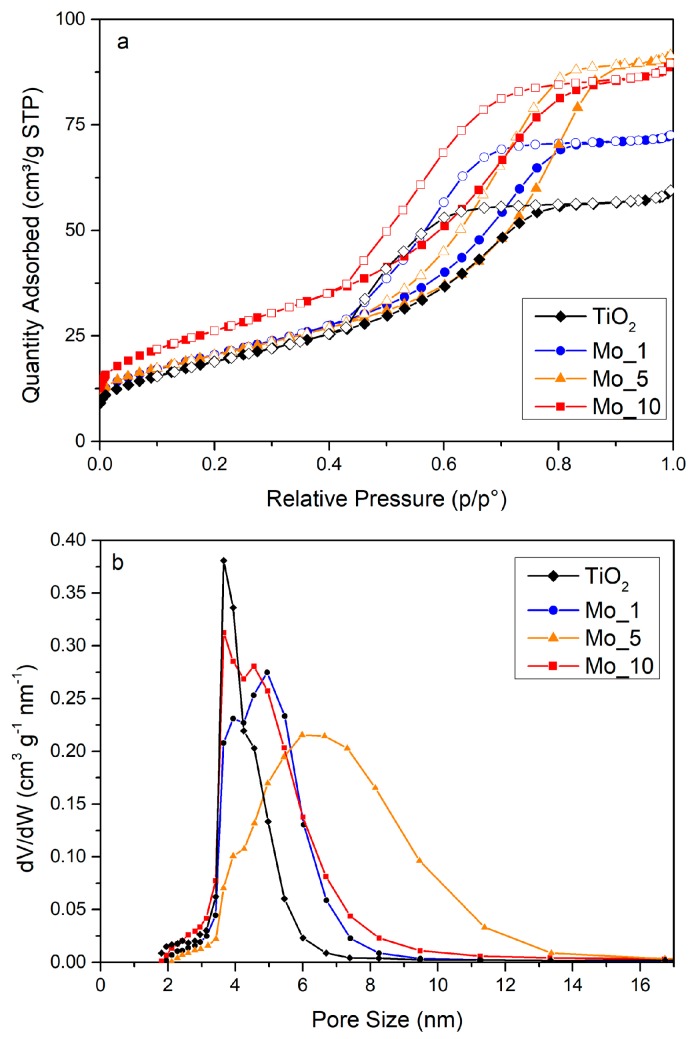
(**a**): N_2_ adsorption/desorption isotherms at −196 °C (full symbols: adsorption branch; hollow symbols: desorption branch); (**b**): pore size distributions (PSDs) of bare and Mo-doped TiO_2_.

Figure 2 shows the N_2_ adsorption/desorption isotherms at −196 °C on both pure and Mo-doped samples, and the corresponding pore size distribution (PSD) as calculated by applying the BJH method to the isotherm desorption branch. All the samples show Type IV isotherms with Type H2 hysteresis loop, due to N_2_ condensation within inter-particles porosity. The BET SSA value (Table 1) was almost unaffected by the presence of Mo for heteroatom loadings up to 5 wt.%, while it increases with the Mo_10 sample.

**Figure 3:**
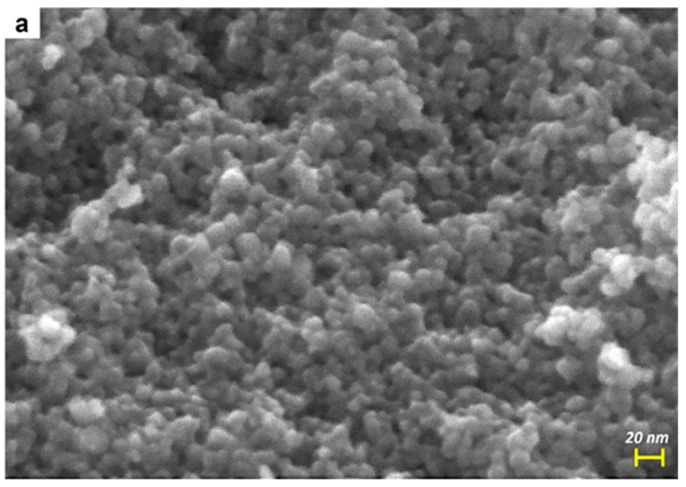

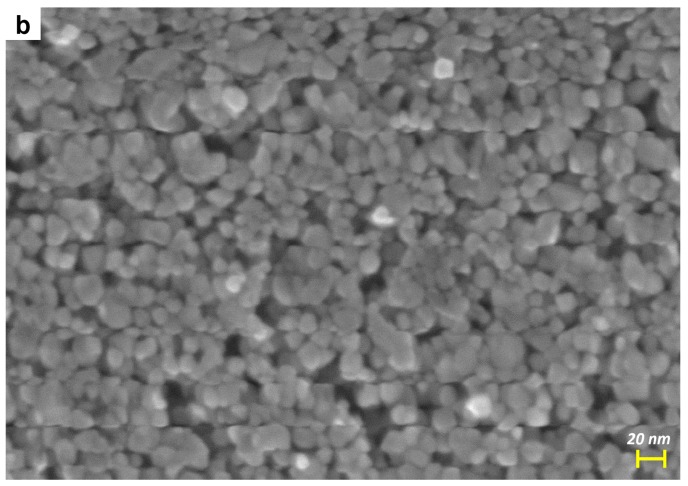
FESEM micrographs of the bare TiO_2_ (**a**) and of the Mo_5 sample (**b**).

The bare TiO_2_ showed a sharp Pore Size Distribution (PSD) in the 3–6 nm range (see Figure 2b), suggesting a homogeneous distribution of particles size. The same sample showed also the smallest particle size (see Table 1) and a quasi-round particle shape (see Figure 3a): from a merely geometrical point of view, the inter-particle porosity is expected to be rather homogeneous with smaller dimensions with respect to the particle size. Interestingly, the PSD became broader with Mo-doped samples. Actually, both the size and shape of the particles could affect the BET SSA and the PSDs, and, probably, less homogeneous (in both size and shape) particles were obtained in the presence of Mo. In particular, the Mo_5 sample showed the broadest PSD (in the 3–14 nm range): the sample showed indeed nearly the same BET SSA of Mo_1 (Table 1), but a larger pore volume. This could be due to the occurrence of NPs characterized by heterogeneous size and shape (see Figure 3b), although some aggregation/agglomeration phenomena may have occurred in Mo_5 as well.

Figure 3a,b report the FESEM micrographs of two selected samples, namely TiO_2_ (a) and Mo_5 (b): NPs with quasi-round shape are observed, but the addition of Mo leads to a slight size increase due to induced structural changes (Table 1) and, indeed, the Mo_5 sample showed NPs with more heterogeneous size and shape, in agreement with the sample PSD (see Figure 2b).

The EDX analysis allowed us determining the Mo/Ti atomic ratios reported in Table 1, showing that in the explored composition range, the actual Mo content was very close to the nominal value.

**Figure 4:**
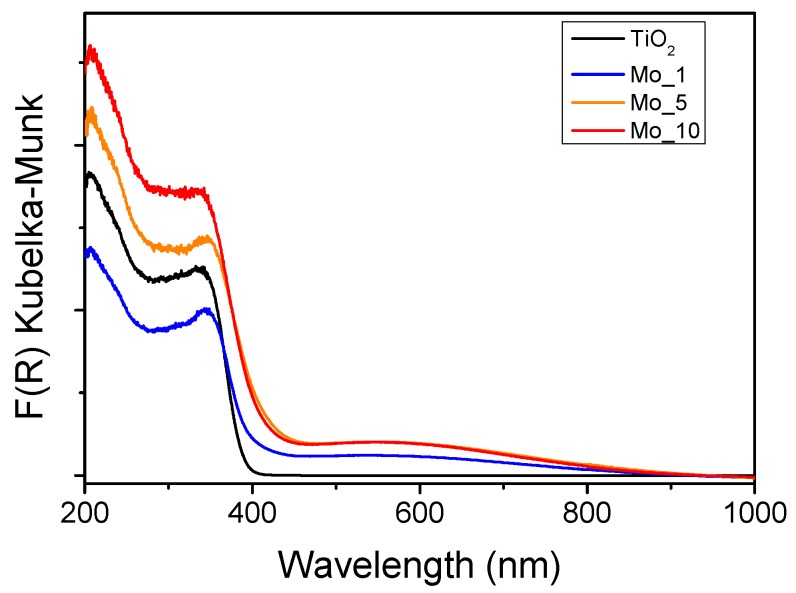
DR–UV–Vis spectra of the samples TiO_2_ (black curve); Mo_1 (blue curve); Mo_5 (yellow curve) and Mo_10 (red curve).

Figure 4 reports DR UV–Vis spectra of the samples. As expected, the TiO_2_ sample absorbed only below 400 nm, whereas introduction of Mo brought about two effects: a slight red-shift of the absorption edge and the appearance of a broad absorption centered at ca. 550 nm readily assigned to sub-band transitions related to mid-band gaps formed by Mo doping [42]. The red-shift of the absorption edge shows that Mo doping is modifying the band gap (E_g_): the corresponding E_g_ values, as obtained by the Tauc’s plot method (not shown), are indeed 3.07 eV (TiO_2_), 2.86 ev (Mo_1), 2.58 eV (Mo_5) and 2.69 eV (Mo_10). Interestingly, the Mo_5 sample showed the lowest E_g_ value: at higher contents, Mo likely tends to form surface species, rather than being dispersed in the bulk, as also confirmed by Raman and XP spectroscopies (vide supra).

**Figure 5:**
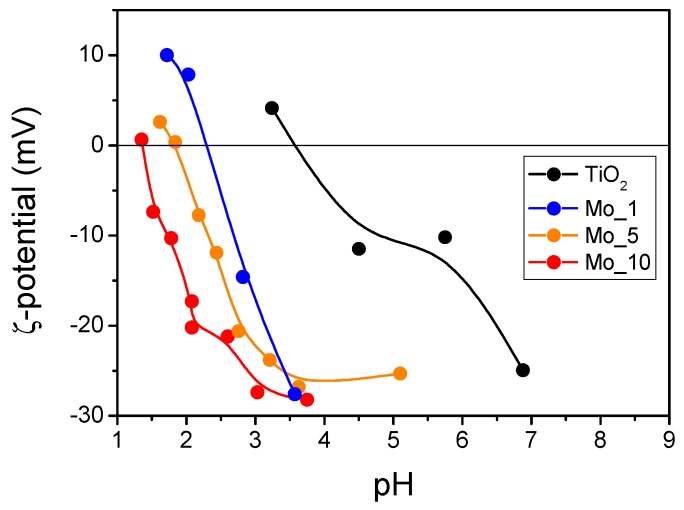
ζ-potential measurement on the samples TiO_2_ (black); Mo_1 (blue); Mo_5 (yellow) and Mo_10 (red).

The ζ-potential measurements (Figure 5) show that the bare TiO_2_ has a Point of Zero Charge (PZC) of 3.6, that is, a value lower than usual for TiO_2_: for instance, Degussa P25 (with particle size between 20 and 40 nm) has a PZC around 6.2–6.9 [43,44]; for pure TiO_2_ NPs (with diameter in the range of 14–33 nm), a PZC of 6.8 [45] was reported; Allard et al. found a PZC of 6.1 with commercial anatase NPs (with a diameter of ca. 20 nm) [46]; Al-Hetlani et al. found a PZC of 5.98 for smaller anatase NPs (around 7.2 nm [47]); and Huijun et al. reported a PZC of 6.2 for anatase NPs (5–10 nm) [48]. The much lower value of PZC reported here may be ascribed, rather than to the size of TiO_2_ NPs, to the type of synthesis [49,50] that probably favors the formation of a very acidic surface, in agreement with previous work [43]. Addition of Mo leads to a further and progressive decrease of the PZC, even at the lowest Mo content (sample Mo_1). Such results confirm that this type of synthesis allows distribution of the heteroatoms not only in the bulk, but also at the NPs’ surface, with some superficial molybdenum ions lowering the PZC, as they are strong Lewis sites.

The ζ potential measurements allowed us to figure out that even at low Mo content, the surface of the NPs is affected by the presence of Mo: the ancillary XPS measurements (Supplementary Information) allowed us to measure the surface Mo/Ti atomic ratio in the studied samples: the corresponding values (reported in Table 1) show that the NPs surface is enriched in Mo atoms with respect to the bulk, in agreement with the type of synthesis adopted.

These obtained samples are negatively charged in a wide pH range (Figure 5), and so they should preferably interact with positively charged species, such as the Rhodamine B (RhB) dye [51].

**Scheme 1:**
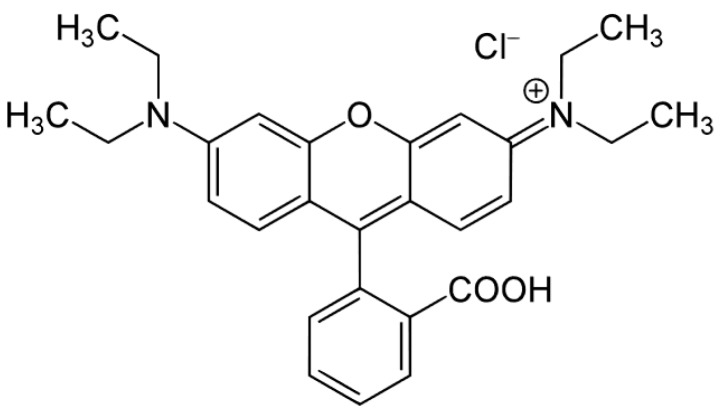
Rhodamine B (RhB).

Indeed, RhB (Scheme 1) is a cationic Xanthene type dye, characterized by the presence of both diethylamine and carboxylic groups and is commonly used as a model dye pollutant [52,53]. For these reasons, the photocatalytic RhB degradation was studied in the presence of the Mo-doped samples and of the bare TiO_2_. Visible light irradiation with a low irradiance value of 33 W/m^2^ was used to avoid or at least minimize any dye-sensitization effect and to test the feasibility of sun-driven photocatalytic reaction [33].

**Figure 6:**
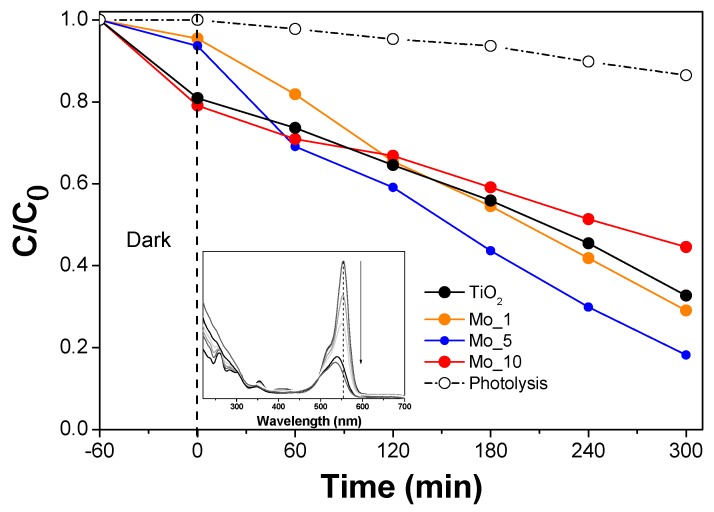
Relative concentration profile of RhB degradation under visible light. Inset UV–Vis absorbance spectra carried out during the photodegradation of RhB in the presence of the Mo_5 sample.

The obtained RhB degradation curves are shown in Figure 6, where, for comparison, the results concerning two blank experiments carried out without any catalyst (mere photolysis) and in the presence of a commercial TiO_2_ sample (Degussa P25) are reported, showing, respectively, that under Vis light, RhD photolysis occurs to a minor extent and that the commercial TiO_2_ was the least active of the tested samples. Concerning the photocatalytic activity of the bare TiO_2_ (E_g_ = 3.07 eV), XRD patterns shows that the sample also contained some brookite (see Figure 1): other mixed TiO_2_ phases containing brookite showed, indeed, promising photodegradation activity towards RhB under Vis light [54]. As compared to the bare TiO_2_, the Mo_10 sample showed similar behavior during the dark step, whereas under illumination, its RhB degradation efficiency was even lower than that of the bare TiO_2_ at a longer reaction time. Notwithstanding its smaller band gap (E_g_ = 2.69 eV) and lower PZC, which should favor, respectively, photocatalytic activity and the interaction with diethylamine groups (protonated in the adopted reaction conditions), the (high) Mo content of the Mo_10 sample likely had a detrimental effect on RhB degradation. At a higher content, Mo tends to form surface species, as shown by both Raman and XPS spectroscopies (the Mo_10 sample has indeed a slightly larger band gap (E_g_ = 2.69 eV) than Mo_5 (E_g_ = 2.58 eV)). Nonetheless, the formation of surface polymolibdates (as detected by Raman spectroscopy) could enhance surface electron/hole recombination, finally decreasing photocatalytic activity. Interestingly, an improved kinetic rate was achieved with the Mo_5 sample, characterized by the smallest E_g_ value (E_g_ = 2.58 eV). Such results suggest that Mo_5 has an optimum dopant concentration allowing a better exploitation of the light (due to the lowest band gap), notwithstanding the adsorption properties of the sample in dark conditions were worse than both TiO_2_ and Mo_10, likely due to the larger particles size of Mo_5 (22 nm, see Table 1). Similar considerations can be drawn for the adsorption behavior of the Mo_1 sample, where the lower Mo content was instead responsible of its lower activity with respect to Mo_5. Concerning the kind of dye–surface interaction, complex phenomena may occur, besides electrostatic attraction between the protonated diethylamine groups of the dye and the surface negative charge of the samples, as RhB also contains carboxylate groups and some positive surface charges may occur, especially when Mo ions are at the surface of the NPs. However, the interaction between protonated diethylamine groups of RhB and the negatively charged surface of the prepared samples could improve the degradation rate by cleavage of the Xantene group. This result could be further confirmed from the shift of the main RhB peak from 554 nm to 535 nm observed in the dye UV–Vis spectrum (inset to Figure 6): such shifts mainly occur when the dye undergoes photocatalytic degradation through a de-ethylation process/route and formation of triethylrhodamine, diethylrhodamine, and ethylrhodamine, with a different λ_max_, at 555, 539, 522 nm, repectively [52,53].

## 4. Conclusions

Both pure and Mo-doped TiO_2_ NPs (with a size of ca. 10–20 nm) were obtained by a reverse micelle sol–gel synthesis method, allowing the dispersion of Mo both in the bulk and at the surface of the NPs, and substantially avoiding segregation of crystalline MoO_3_ event with 10 wt.% Mo.

The 5 wt.% Mo content was found to provide an optimal lowering of the band gap (from 3.07 V for the bare TiO_2_ to 2.58 eV), which resulted in the fastest kinetics during the photocatalytic degradation of the model dye Rhodamine B (used here as a characterization technique).

The synthesis also led to a very acidic (polar) surface, even in the absence of Mo: the resulting NPs were indeed negatively charged in a wide pH range. This surface negative charge, however, did not enhance the degradation of the dye studied here, especially at the highest Mo content, because the surface polymolibdate species likely acted as recombination centers of electron/hole pair. Despite all this, the high acidity of the NPs surface could be exploited in applications requiring a very polar surface, due to the possibility to polarize some organic pollutant, finally promoting its adsorption and consequent degradation.

## Figures and Tables

**Table 1:** Textural and surface properties of the studied samples as determined by N_2_ isotherms at −196 °C; FESEM; XRD, EDX and XPS (X-ray Photoelectron Spectroscopy) analyses.

Sample	BET SSA (m^2^ g^−1^)	Total Pore Volume (cm^3^ g^−1^)	Average Particle Size (±s.d. nm)	Average Crystallite Size (±s.d. nm) *	EDX Determined Mo/Ti (Nominal Mo/Ti) Atomic Ratios	XPS Determined Surface Mo/Ti Atomic Ratio [32]
TiO_2_	71	0.091	12 (3)	10.3 (0.5)	-	-
Mo_1	76	0.112	21 (5)	12.5 (0.9)	0.0070 (0.0084)	0.042
Mo_5	74	0.141	22 (5)	12.5 (0.4)	0.05 (0.044)	0.150
Mo_10	96	0.137	18 (4)	9.9 (0.5)	0.090 (0.092)	0.194

* Calculated anatase crystallite size as obtained according to the Williamson-Hall method.

## Supplementary Materials

The following are available online at https://www.mdpi.com/1996-1944/12/6/937/s1, Figure S1: EDX spectra of the studied samples, Figure S2: XPS survey spectra of the studied samples [41].
