# Supplementary material for: Application of Reverse Micelle Sol–Gel Synthesis for Bulk Doping and Heteroatoms Surface Enrichment in Mo-Doped TiO2 Nanoparticles
Source: Materials (Basel). 2019 Mar 21;12(6):937. doi: 10.3390/ma12060937 (PMC6471443; doi:10.3390/ma12060937)
Supplement: Supplementary file 1 [file materials-12-00937-s001.pdf]

materials

Supplementary Information

Application of Reverse Micelle Sol–Gel Synthesis for Bulk Doping and Heteroatoms Surface Enrichment in Mo-Doped TiO_2 Nanoparticles

Roberto Nasi [1], Serena Esposito [1], Francesca S. Freyria [1], Marco Armandi [1], Tanveer A. Gadhi [2], Simelys Hernandez [1,3], Paola Rivolo[1], Nicoletta Ditaranto[4] and Barbara Bonelli [1,5,*]

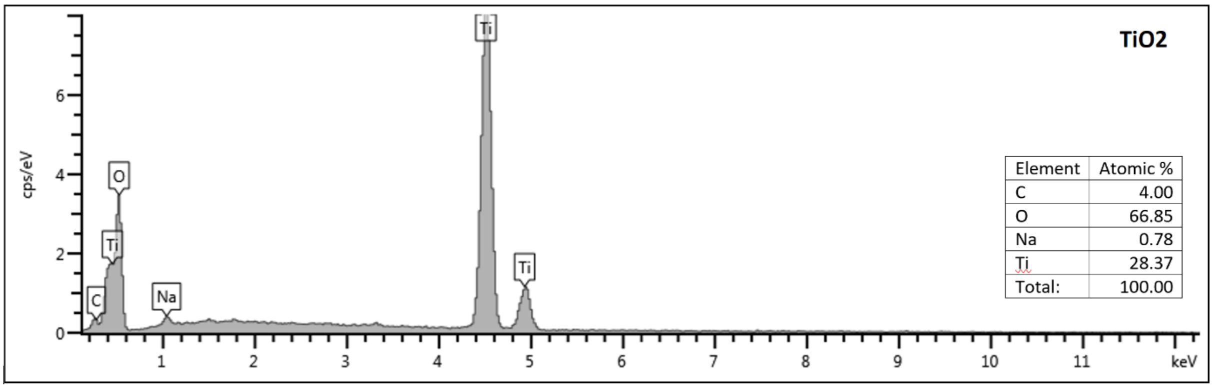

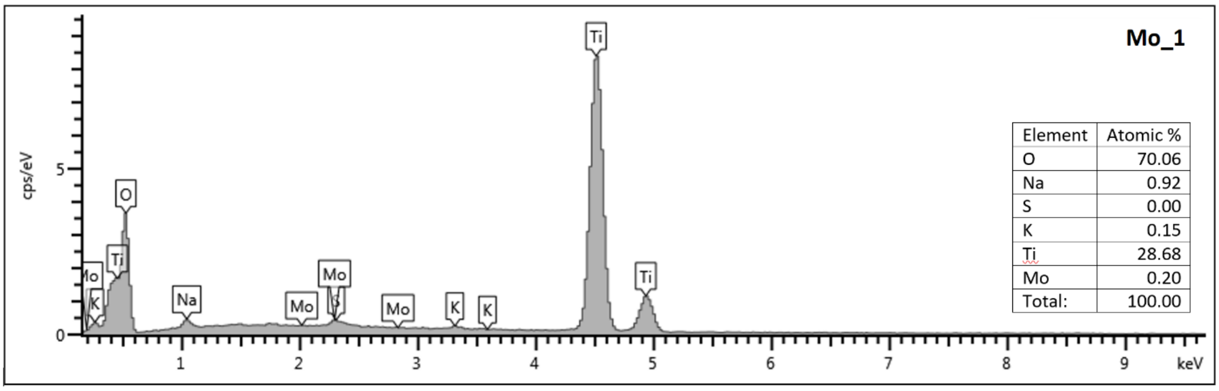

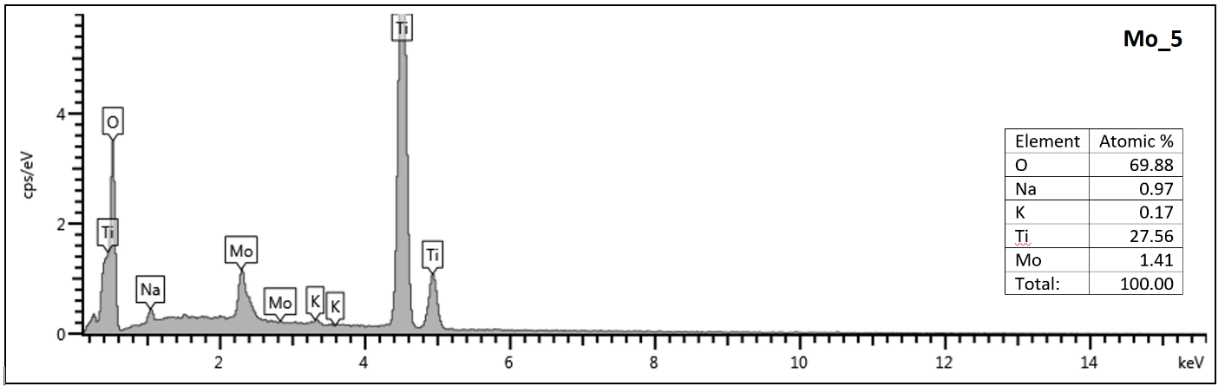

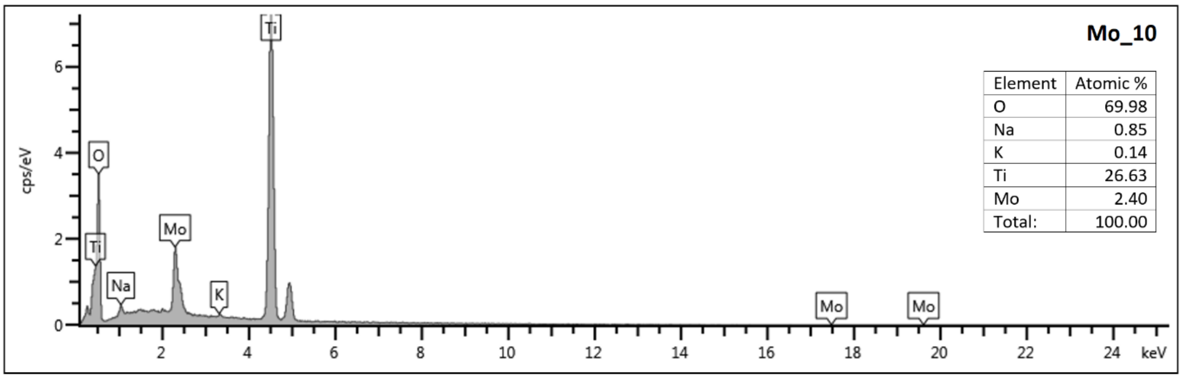

Figure S1. EDX spectra of the studied samples.

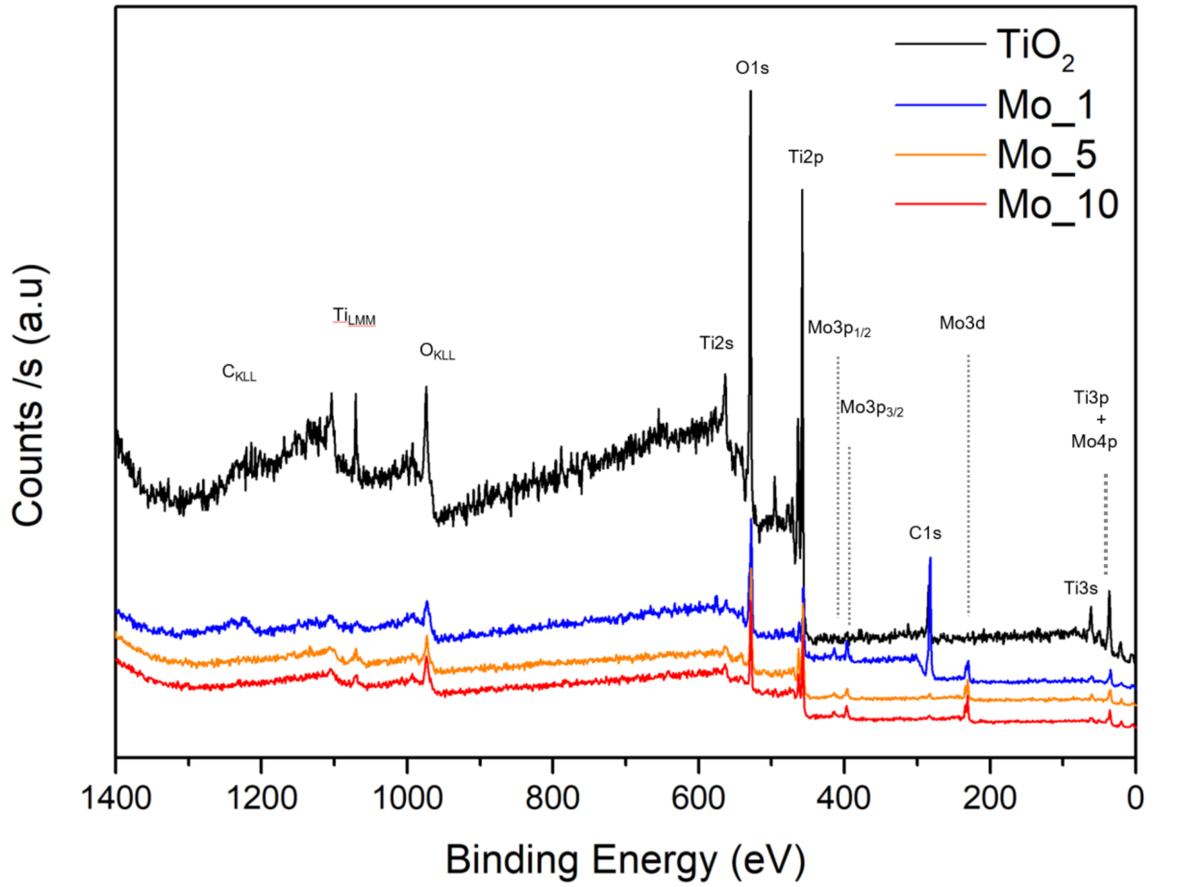

Figure S2. XPS survey spectra of the studied samples [41].
